# Raising an Assistance Dog Puppy—Stakeholder Perspectives on What Helps and What Hinders

**DOI:** 10.3390/ani10010128

**Published:** 2020-01-13

**Authors:** Dac Mai, Tiffani Howell, Pree Benton, Pauleen C. Bennett

**Affiliations:** 1 Anthrozoology Research Group, Department of Psychology and Counselling, School of Psychology and Public Health, La Trobe University, Flora Hill, VIC 3552, Australia; t.howell@latrobe.edu.au (T.H.); pauleen.bennett@latrobe.edu.au (P.C.B.); 2 Centre for Service and Therapy Dogs Australia, Melbourne, VIC 3162, Australia; Pree.Benton@dogsforlife.com.au

**Keywords:** puppy raising, program adherence, program satisfaction, organisational performance, program engagement

## Abstract

**Simple Summary:**

Puppy raisers (or foster families) are volunteers who care for assistance dog puppies until they are ready to learn how to help people with a disability. During this period, some puppies develop behaviours unsuitable for assistance roles and end up changing careers or being rehomed as pets, which is wasteful. Puppy raisers control the early experiences of their puppies, and they vary in their puppy-raising outcomes, but we do not know what specifically helps some puppy raisers produce puppies that are behaviourally suitable for an assistance role. In this study, we interviewed 17 people from seven countries who were either experienced puppy raisers or provider program staff, or both. Their responses suggested several individual factors (*expectations*, *competency*, *perseverance and passion*) and social factors (*informational* and *emotional* supports), in addition to the puppies’ characteristics, that influenced the experiences and perceived effectiveness of their puppy-raising practices. These factors are also evident in other well-established areas of research (e.g., education, volunteerism, social support, and organisational performance). We propose recommendations for assistance dog organisations based on those relevant frameworks, which focus on enhancing puppy raisers’ competency, positive experiences, and program retention.

**Abstract:**

Assistance dog puppies live with their raisers for up to 16 months before entering advanced training and, hopefully, becoming qualified to help people with a disability. Almost half of the puppies fail to meet the behavioural standards required for assistance dogs, and some puppy raisers produce more behaviourally favourable puppies than others. It is unclear what factors influence puppy-raising practice quality. To understand this, we interviewed 17 participants, including experienced puppy raisers (*n* = 8), provider organisation staff (*n* = 4), and those who have served both as puppy raisers and staff (*n* = 5). Results of a thematic analysis suggest three groups of influencing factors, namely *intrapersonal factors*, *social support*, and *puppy characteristics*. Intrapersonal factors such as *expectations*, *competency*, *perseverance and passion* were reported to influence puppy raisers’ experiences, puppy-raising quality, and continuity of service. Contextual factors such as availability of social support (*informational* and *emotional supports*) and less-demanding puppies both led to positive puppy-raising experiences, while the former also contributed to puppy raisers’ perceptions of competency. Future research should quantitatively examine the interrelationships of these factors concerning puppies’ behavioural development. Meanwhile, organisations could consider these factors when developing their recruitment and puppy-raiser support programs.

## 1. Introduction

There has recently been an increase in demand for assistance dogs; dogs specially trained to help individuals with a disability such as visual, hearing, mobility impairment, autism spectrum disorder, and or post-traumatic stress disorder [1,2]. Many organisations breed or purchase puppies for this purpose. Before being trained and assessed for their ability to safely function in various places and situations, these puppies must be reared, starting as young as six to 12 weeks [3], for up to 16 months before they are suitable for advanced training, which prepares them for their specific work role. Puppy raisers, typically volunteers recruited either from the public [3] or from institutions such as higher education and correctional settings [4,5], provide care and opportunities for the puppies to learn and become familiar with a wide variety of environments and situations. 

Although assistance dog provider organisations generally oversee the puppy-raising practices of their volunteer raisers, research has found that puppy-raising outcomes vary widely amongst puppy raisers [6,7,8]. Although very little information is available about graduation rates for assistance dog puppies in general, it has been reported that approximately half of all guide dog puppies fail to qualify and work in their intended role [1,9,10]. This high failure rate is unacceptable for various reasons, such as economic inefficiency and welfare of the dogs after being disqualified and rehomed [11]. It is of interest, then, that one primary reason for failure is dogs having behaviours unsuitable in public places, defined broadly as behaviours associated with aggressiveness, distractedness, stress and fearfulness [12,13]. Experiences during the raising period are critical to puppies’ behavioural development [14,15,16], and could help reduce puppies’ anxiety and fearfulness if appropriately managed [17]. Since raisers have a high degree of control over their puppies’ experiences during this time, it is essential to understand what helps and what hinders the practices of puppy raisers.

Research has found that puppies’ behavioural outcomes vary among puppy raisers and in favour of those with more experience. Serpell and Duffy [6] found that puppies of raisers who had raised multiple puppies were less aggressive and less fearful when encountering strangers or unfamiliar dogs. Body sensitivity to touch, an undesirable trait generally associated with avoidant behaviours, was also higher in puppies raised by less-experienced puppy raisers [6]. It is unclear, however, exactly why experienced puppy raisers produce more favourable outcomes.

It could be that multiple-time puppy raisers have had more opportunities to acquire knowledge and practice relevant puppy-handling skills, which leads to an increase in their competency. Fratkin [18] reports that experienced puppy raisers rated themselves more highly on the understanding of puppies than inexperienced puppy raisers. Additionally, puppies raised by raisers who self-reported as more knowledgeable and more experienced were also rated significantly more favourably by their raisers on traits relating to dogs’ attachment to their raisers, and trainability [18]. These differences are noteworthy and suggest the formation of secure attachments by the dogs to the raisers. Attachment styles are well established in human relationship research and refer to how a person reacts to the demands in their relationships, with characteristic styles typically developing in infancy [19,20,21]. Research in dog–human relationship suggests similar patterns of attachment to those between children and their parents [22]. Dogs with a secure attachment style are more confident to independently explore novel objects in the presence of their owners [22,23]. Conversely, dogs with an insecure attachment style constantly seek proximity to their owners and typically display signs of distress during separation from their human attachment figures [22,24]. Given the evidence that children receiving a high quality of care during infancy exhibit higher rates of secure attachment [25], it may be reasonable to expect differences in attachment styles in puppies in Fratkin’s [18] study as they were raised by raisers with various levels of experience and understanding of puppy raising.

Literature indicates that behaviours associated with insecure dog-owner attachment types (e.g., seeking attention from handlers, becoming agitated when owners show affection to other people, dogs or animals), and/or lower trainability (e.g., less responsive to commands, and less controllable when in public spaces) [26] are generally considered undesirable in existing research on assistance dog puppy raising [16,27]. An alternative explanation for why multiple-time puppy raisers produce better puppy outcomes could be that puppy raisers who are successful at puppy raising the first time around are more likely than others to continue raising subsequent puppies. In either case, successful puppy raisers should ideally be encouraged to continue to raise subsequent puppies to improve success rates and outcomes for assistance dog organisations and their future handlers. Therefore, the organisation plays a vital role in ensuring positive experiences for, and enhancing retention of, puppy raisers.

Concerning program engagement and satisfaction, insufficient instructions for effective puppy-handling techniques and lack of technical support were found to be detrimental to the experiences of first-time puppy raisers [28]. Chur-Hansen, Werner, McGuiness and Hazel [28] found that lack of preparation and organisational supports impaired first-time puppy raisers’ experiences in such aspects as their psychological, physical, and social well-being. These researchers interviewed nine first-time puppy raisers at one guide dog provider organisation, across four time points (i.e., before the puppy’s arrival, and at week one, month three and month 13). In the first interviews, respondents reported high expectations for their puppies and anticipated that they would receive positive puppy-raising experiences. However, at the latter three time-points, they repeatedly reported struggling with their puppy’s undesirable behaviours and experienced resentment regarding a lack of support and responsiveness from the organisation. These participants reported a need for support, including: more preparation before their puppy’s arrival; a more accurate description of the required workload; more training and information, feedback and reassurance from the organisation; and access to a support group. Another common feature was that puppy raisers loosened the rules of the organisation in times of difficulty. Lack of organisational support, therefore, appeared to affect not only the puppy raisers’ experiences but also their tendency to strictly adhere to puppy-raising instructions.

Lack of organisational guidance leaves puppy raisers in a position where they have to rely on their own ideas about puppy handling techniques, which may not be suitable [29]. Koda [29] conducted a study to explore how puppy raisers coped with puppies’ undesirable behaviours when not receiving any formal guidance on puppy raising, except for a request to not physically punish the puppies. Although the program in Koda’s study was provided by a guide dog training organisation, the participants were informed that the lack of formal instruction was part of the design of the study and not the provider’s standard practice, and that their puppies would not become future guide dog candidates. Observing at-home practices, Koda found that puppy raisers resorted to personal techniques (e.g., ignoring or distracting a puppy) to stop undesirable behaviours. However, most of the techniques they used were not effective at addressing their puppy’s problem behaviours (e.g., biting, vocalising, damaging household items).

These findings suggest that a lack of support and training in effective puppy handling and training techniques is likely to lead to less than optimal puppy-raising outcomes. While this situation, in which raisers were not provided with any guidance at all, may be unlikely in practice, it is clear that puppy raisers’ practices vary depending on their experience [6,7,8] and appear to be affected by levels of organisational support and guidance [28,29]. In this study, we aimed to further explore what is helpful and what needs to be improved to enhance puppy raisers’ experiences and their ability to engage in optimal puppy-raising practices.

## 2. Materials and Methods

The La Trobe University College of Science, Health and Engineering Human Ethics Sub-Committee approved this study (HEC 18-381) on 3 October 2018.

### 2.1. Participants

Seventeen participants were recruited for this study from 11 assistance dog provider organisations, across seven countries (see Table 1). Inclusion criteria were being 18 years or older, having raised one puppy or being a staff member responsible for a puppy-raising program at an assistance dog organisation, and being able to speak English. Twelve of our participants functioned either as puppy raisers (*n* = 8) or staff (*n* = 4). One participant (SR09) had been a staff volunteer for five years before raising her first puppy at the time of the interview. The other four were puppy raisers serving as staff or mentors/counsellors for other puppy raisers; one of them (SRF17) was the founder and head trainer of their organisation and was the person who bred and raised all of the puppies, with support from volunteers and other trainers. The organisations represented train and provide dogs for various assistance roles including, but not limited to, guide dogs, hearing dogs, and mobility dogs.

### 2.2. Materials

Interviews were guided by two semi-structured interview schedules (see Table S1), one for puppy raisers and another for staff. A selection of questions from both versions guided interviews with experienced puppy raisers who also served as staff or mentors/counsellors. Initial questions asked about the respondent’s experience (for puppy raisers) or perceptions (for staff) of the puppy raiser role, and what contributed to successful puppy raising. Other questions were designed to explore what was helpful and challenging about puppy-raising practices. Where applicable, references to difficulties experienced by respondents when raising their first puppy were followed up with questions such as (1) what they did, (2) what their organisation did, or (3) what, if anything, they believed should have been done differently in response to those situations.

### 2.3. Procedure

A total of 143 organisations from 19 countries, of which contact details were publicly available on their websites, were sent an email with information about this study, with a request to notify their staff or puppy raisers about the study. Interviews were conducted via teleconference over five months from November 2018 to March 2019, with lengths ranging from 30 to 70 min. All interviews were recorded and transcribed verbatim. Participants then received their transcriptions to check for accuracy and suggest any modifications. The data set was then analysed using QSR International’s NVivo 12 qualitative data analysis software [30].

### 2.4. Data Analysis

An inductive content analysis [31] was used to condense data into a meaningful conceptual model. Data collected were validated from both the participants’ and the researchers’ perspectives as per Creswell and Miller [32]. From the lens of the participants, data were validated through member checking, whereby we returned the interview transcripts for affirmation and modification [32]. On the researchers’ side, validation was conducted through triangulation, a procedure whereby data are collected from different sources to investigate a topic [32,33]. The current study collected data from participants with different roles, such as puppy raisers, staff, and puppy raisers serving as staff/mentors. We also recruited participants from different countries and from different organisation types (e.g., guide dogs, hearing dogs, mobility dogs). This enabled us to explore a wide range of experiences and aspects of puppy raising, allowing for cross-validation of the emerging themes, and ensuring the data were enriched and our understanding deepened by inclusion of multiple perspectives [33,34].

Data were analysed thematically as per Braun and Clarke [35]. Emerging themes were validated via extensive discussions among the authors, as well as with another researcher who is proficient in using qualitative methods in assistance dog research and was independent of this study. Throughout the results section, direct quotes are presented to give voice to the participants. However, to aid readability, participants’ repetitions and filler words (e.g., ‘um, well, um…’) are removed. Where appropriate, additional information in parentheses provides contextual explanations, whereas square brackets indicate grammatical corrections and replacement of identifiable information.

## 3. Results and Discussion

The thematic analysis suggested three categories of factors affecting puppy-raising practices, namely: intrapersonal factors, social support, and puppy characteristics.

### 3.1. Intrapersonal Factors

#### 3.1.1. Expectations

During first-time puppy raising, having unrealistically high expectations for the puppy’s training progress was reported as unhelpful, resulting in negative experiences for the puppy raiser. One puppy raiser (R05) expressed that they were “*very afraid of making mistakes [and] very worried that [their puppy] wouldn’t be good enough*”. One staff added to this saying that “*[puppy raisers] put more pressure on themselves sometimes than [their organisations] even do to get things right*.” (S15).

Most puppy raisers reported having more reasonable expectations after raising their first puppy. One puppy raiser narrated:

“*I think because she was going to become a mobility dog, I had quite high expectations of her behaviour when she was really young that I don’t have any more with so many other puppies coming through. I still do have high expectations of them, but probably not as unrealistic as they were when the first puppy came through. Because they’re growing into a mobility dog, you almost expect them to be little mini mobility dogs when they arrive and behaving more reasonably than a little puppy does usually, the tiny puppy that usually runs around and wants to have fun.*”

(R06)

The reported adverse effects of having unrealistically high expectations on puppy-raising experiences in the current study were in line with the literature in organisational research [36]. Job expectation refers to what employees believe they could achieve on that job, whereas realistic expectations concern the expectations that they could eventually confirm as being met [37]. Having unrealistically high job expectations, which are likely unmet, results in negative attitudes in employees towards their jobs [36]. On the other hand, new employees with more realistic expectations were found to have higher job satisfaction and lower turnover rates [38,39,40].

Although this indicates that it would be beneficial for organisations to provide their prospective puppy raisers with a thorough description of the puppy raiser role, merely receiving information about puppy raising may not result in development of realistic expectations. Research on social learning in other contexts has found that individuals who observed or received information were not as realistic in judging the difficulties of tasks as those who performed them [41]. This confirms that information is not a substitute for experience. Although it is beneficial for organisations to provide information to help their first-time puppy raisers form more realistic expectations of this role, a complementary strategy might be to offer a range of short-term fostering experiences, such as supervised weekend puppy sitting for puppies of various ages and states of training, before asking puppy raisers to sign up for a year-long role. While logistically challenging, this preparation period could be a prelude to accepting a volunteer as a puppy raiser. This would not only provide interested volunteers with realistic expectations about various stages of the puppy-raising role but also allow organisations to assess if their volunteers have acquired the necessary skills and knowledge to handle and train puppies at different developmental stages.

#### 3.1.2. Competency

Not surprisingly, puppy-raising competency enables puppy raisers to respond effectively to their puppy’s issues, while lack of competency negatively affects their ability to raise their puppy effectively. Most participants reported the importance of raisers having adequate knowledge and skill levels necessary to raise puppies to the quality required of a trained assistance dog. Lack of competency also negatively affected their experience. One puppy raiser recalled “*I remember the first few months being quite difficult because I wasn’t sure what I was supposed to do.*” (R06).

Several other participants echoed this sentiment, suggesting that first-time raisers are particularly disadvantaged and that raising subsequent puppies is less stressful because raisers can anticipate challenges in advance and have plans in place to deal with their puppy’s issues. One participant (SR09) who had volunteered as staff for five years prior to raising her first puppy still reported challenges when raising a puppy for the first time:

“*As a first-time raiser, I’m learning as much as the puppy is. I didn’t know how to handle it right. If I were raising a second time, I’d be a much better puppy raiser. I think my puppy would have learned things quicker, or earlier at least.*”

(SR09)

**Figure 1:**
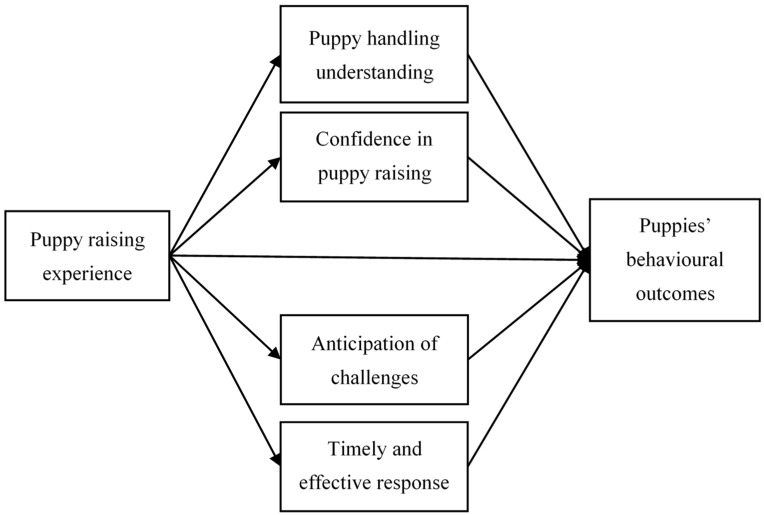
Competency mediates the relationship between raisers’ experience and puppies’ behavioural outcomes.

Lack of puppy-raising understanding, particularly the ability to anticipate what is involved in raising assistance dog puppies, clearly negatively affects experiences and practices of first-time puppy raisers. These findings may explain previous results demonstrating better behavioural outcomes [6] and higher self-rated puppy-raising understanding [18] amongst experienced puppy raisers. Our findings suggest a causal pathway, shown in Figure 1, in which experience increases raisers’ understanding of puppy behaviour, enabling them to feel more confident, predict challenges, and respond to them more quickly and effectively, thereby improving outcomes.

While this pathway looks straightforward, it is important to consider whether any prior experience with dogs will suffice or whether specific experience in raising an assistance dog puppy is necessary. For first-time puppy raisers without prior experience, another consideration is whether the knowledge typically gained via experience can be acquired in another way, to better prepare them for the task ahead. Allowing puppy raisers to raise their first puppy unsuccessfully, purely to gain the experience required to raise a second puppy successfully, would be very wasteful and should not be necessary. After all, we expect parents to raise their first child successfully, even though contemporary communities provide varying levels and types of supports to assist in this aim.

Participants reported that prior experience in dog obedience training was especially beneficial when the previous training methodologies agreed with those of the organisation supplying the puppy. One participating program coordinator (S15) attributed positive progress in their program to puppy raisers’ competency in obedience and their prior experience with addressing dogs’ behaviours. When asked about the effects of using shared training techniques on their puppy raising, one puppy raiser stated:

“*Their [training] philosophy and my philosophy is the same. At the end of the day, we’re both trying to achieve the same outcome, which is to produce a dog that’s suitable for training to be a guide dog for someone. So, I have no troubles at all with following their advice or talking to them.*”

(R02)

Prior experience with an opposing technique (correction-based versus reward-based), in contrast, may interfere with the puppy raisers’ process of learning the techniques used by the organisation. A couple of staff reported experiencing some difficulty when teaching puppy raisers whose cultural or existing personal dog-handling techniques did not agree with those of the organisation. One puppy raiser coordinator explained:

“*We … strictly train by positive reinforcement. So, people don’t always know of [these] techniques, [and] getting them to unlearn what they may have learned as kids or with [their] own pet dogs, has been more difficult. So, that is where we struggle the most.*”

(S13)

The influence of puppy raisers’ prior knowledge on their practices and their capacity to learn a new approach is in line with a constructivist view of learning [42]. According to this paradigm for adult learning [42], prior knowledge not only provides a foundation on which new understanding is formed but can also interfere with the knowledge acquiring process. Existing knowledge and experiences are activated and compared when encountering new ideas and knowledge. High similarities of current understanding enhance retention of the novel learning, whereas contradicting prior knowledge hinders the learning process [43].

A problem for puppy-raising organisations, then, is a general lack of consistency in dog training methods used in the community, which has been a topic of discussion in the literature concerning companion dog owner populations [44,45]. Todd [44] suggests that a lack of knowledge and regulation in the dog training industry poses barriers to dog owners adopting humane training techniques. Similarly, Feng, Howell and Bennett [45] found that it takes specific skills and knowledge to efficiently use clicker training, a reward-based dog training method, and that most of those who endorsed this method were professional dog trainers and trained dog owners. Dog owners with limited experience in dog training, on the other hand, were more likely to adopt dog training methods other than clicker training, including reward-based methods such as verbal praise and patting, and verbal and physical punishment [45]. This situation seems unlikely to change in the foreseeable future. Therefore, puppy-raising organisations should be strongly encouraged to restrict recruitment of puppy raisers to those people who have experience with compatible dog training techniques, or who at least express a willingness to embrace the required approach.

#### 3.1.3. Barriers to Help-Seeking Behaviour

A lack of awareness about puppy raising was unhelpful to puppy raisers. Reflecting on their first-time puppy-raising experience, one puppy raiser (R08) reported that, although they were aware of the availability of supports, a lack of understanding of when to seek help left them feeling unsupported. This puppy raiser suggested: “*[Organisations should] tell us things that we don’t even know to ask, for first time raisers. After that, I think we [would be] a little more relaxed, or we know when to reach out for help.*” (R08).

Perceived judgement from others was also a barrier to seeking help and negatively affected inexperienced puppy raisers’ learning. In the current study, although puppy raisers reported being aware of different types and sources of support available to them, being afraid of judgement about their puppy-raising performance resulted in hesitation to reach out for help and advice. One puppy raiser said: “*I know raisers in my group were always like ‘well I don’t wanna bring that up because then you’ll think that I’m not doing a good job’*” (RS12). To address a similar issue, one program supervisor described what their organisation had done:

“*We’ve tried to really create a safe environment so that it’s okay to ask questions. We’re always going to be there to help and support you. We’re not going to criticise or tell you that you’re wrong or that sort of thing. So, even in our sessions that have first-time raisers, [they] are willing to get up and to try something, which they wouldn’t do that if they didn’t feel safe. That’s really important to us.*”

(RS10)

The influences of perceived judgement on help-seeking behaviours have been a research focus in a higher education setting [46,47]. Grayson and colleagues [47] found that perceived judgements that threatened self-esteem kept tertiary students from seeking academic advice from their tutors and lecturers. It was also noted, however, that there was a tendency to reach out to instructors for assistance if students perceived potential negative judgements for not seeking help. This latter finding suggests a possible way to promote puppy raiser’s help-seeking behaviour—by expressing to them that there may be some disadvantages for not seeking help, rather than for seeking help unnecessarily or inappropriately. Reported efforts to eliminate negative attitudes towards help-seeking, described above in the quote from a participating program supervisor (RS10), appear to demonstrate that this approach may have merit in the context of puppy raising.

#### 3.1.4. Perseverance and Passion

As discussed previously, it is in the best interests of assistance dog organisations that puppy raisers should continue raising puppies as they gain competency and develop more realistic expectations. One condition of this is that they need to work through the process of raising their first puppy, despite the presence of challenges. A second condition is that they need to learn from this experience, to develop appropriate competencies. When examining the effects of a construct known as ‘grit’ on the attainment of challenging undertakings, Duckworth*,* et al. [48] found that sustained perseverance and passion contributed more to success than intelligence or personality type. According to these authors, perseverance refers to the continuity of practice, while passion refers to positive attitudes towards engagement in that practice. Although these two dimensions are conceptually distinct, the former is often conditional on the latter.

Still, most of us can identify examples where passion and perseverance are not sufficient to develop expertise. In the current study, participants frequently referred to a sense of achievement as a source of motivation or passion to persevere with puppy raising. This sense of achievement was associated with being able to see progress towards achieving the goal of raising a high-quality dog, which is a driving force to help puppy raisers proceed through the long process of puppy raising and also to progress to a second or third puppy. Presumably, puppy raisers who are passionate and persistent but who fail to achieve the required competencies would be less likely to continue, although organisations might do well to remove volunteers who continually produce poorly behaved dogs from their pool, even if this risks bad publicity.

Most of the participating puppy raisers reported finding it helpful to engage in self-motivation and to reassure themselves to keep pushing through difficult times during their first-time puppy raising. One puppy raiser (RS12), who mentored other puppy raisers, suggested that “*[what we should do when experiencing] difficulty is taking [a] deep breath and go okay everything [is] fine. You know, we’re just [going to] proceed calmly with the thing we need to do and get on with it.*”

In contrast to a sense of achievement, there was one reported factor related to passion for the puppy-raising process, which could potentially hinder a raiser’s intention to deal with challenging behaviour and to set the puppy up for success. This was a high attachment to their puppy. One experienced puppy raiser and mentor shared:

“*[It’s] always in the back of your head [that] you’re going to have to give this dog up. In the beginning, you’re like: ‘Oh yeah, I can do it.’ As time goes on, you’re like: ‘Oh man, is there something I can do to mess this dog up, so it doesn’t make it?’ I mean, there’s an emotional component there.*”

(RS10)

One might hope that, in practice, very few puppy raisers would actively jeopardise their puppy’s training so they could later adopt it. Nonetheless, attachment to a puppy and a reluctance to relinquish it may be demotivating in terms of addressing the puppy’s undesirable behaviours, reducing the strength of the raiser’s good intentions. Regarding the concept of grit, this might mean that the raiser lacks the necessary willingness to persevere with difficult tasks and may subsequently be less likely to raise any future puppies.

Another factor that may affect volunteers’ sustained motivation during puppy raising is differences in their reasons for participating in a puppy-raising program. Clary, et al. [49] suggest that people vary in their motivations to participate in voluntary work. Reported motivations to be a puppy raiser in the current study were not limited to helping other people, but also extended to learning dog training skills, trialling dog-ownership before acquiring a family dog, and a desire to have a dog or a puppy.

According to Self-Determination Theory (SDT) [50], the above reasons are autonomous motivations, with sources internal to the person [50]. These contrast with controlled motivations, the external sources of reward and punishment that regulate one’s behaviours [50]. Controlled motivations may be less relevant to the context of puppy raising because this practice is primarily voluntary. Autonomous motivations have been found to predict incremental work efforts and high program engagement amongst volunteers in other contexts [51,52,53]. In the context of puppy raising, the desired types of autonomous motivations favouring a puppy’s success could be to provide assistance dogs to help other people or to improve their own dog training competency. Although, some puppy raisers’ initial motivation may be to enjoy puppy companionship, this may not be hindering so long as they also have autonomous motivations to produce high-quality puppies. Given the previously discussed negative impact of raisers having a strong attachment with their puppies, it is then advisable that organisations promote and reinforce the desirable kinds of motivations in their puppy raisers. A way to do this may be by providing prospective puppy raisers with information about the positive impact of their puppy raising on both their puppy and the future handler.

In summary, having prior experience in puppy raising understandably enables puppy raisers to raise and train their puppies more successfully. In the absence of prior experience in assistance dog puppy raising, experience with general dog training can be helpful, provided the preferred training technique is in harmony with the techniques and philosophy of the puppy-raising organisation. Where prior experience is not available, puppy raisers must be encouraged to access appropriate supports, which requires providers to promote appropriate attitudes toward help-seeking behaviours, making them feel less negatively judged for accessing supports than they are for not accessing supports. Novice puppy raisers typically lack awareness of potential problems and therefore find it challenging to know when support is needed. Other factors that appear instrumental in terms of promoting good practice are motivations and passion for achieving the desired outcomes, and a puppy raiser’s ability to persevere during challenging events. Predicting puppy-raising challenges and providing appropriate information and support before these occur is likely to be a critical process for puppy-raising organisations.

### 3.2. Social Support

The above analysis confirmed the generally demanding nature of puppy raising, especially for novice puppy raisers [28], whose strategies to cope with puppies’ development of undesirable behaviours may be ineffective [29]. It also highlighted the importance of puppy raisers receiving appropriate support. In the current study, all puppy raisers reported receiving different types and degrees of support from both staff and other puppy raisers at their organisation as well as people external to the organisation. These various supporters were reported to have helped the puppy raisers by providing: (1) informational support, including formal instructions, informal sharing of advice or information, or direct assistance with training and socialising; and (2) emotional support, including reassurance and sharing of experiences and responsibility for puppy raising.

#### 3.2.1. Informational Support

Participants from different organisations reported receiving various forms and amounts of informational support. What was consistent was the reported advantage of receiving information in multiple modalities, to meet different types of needs and learning styles of puppy raisers. One illustration of this was different attitudes observed towards puppy-raising manuals, the most popular form of informational material received by participants. Most puppy raisers suggested that manuals provided them with relevant information, particularly when they were written in simple language and did not require prior foundational knowledge. On the other hand, one puppy raiser (R08) revealed that having many things to read overwhelmed them when they raised their first puppy.

Some puppy raisers had several options for obtaining information about puppy raising, such as a telephone hotline and in-person support. One puppy raiser explained that “*if you can’t find the answer in there (the manuals), you can always call someone at [the organisation] in the puppy development (department) and they can try to help you over the phone.*” (RS11). Other forms of information were also available to puppy raisers to ensure they could find answers they needed, such as video demonstrations, an online library, videoconferencing or in-person support during training sessions, home visits and occasional appointments.

In some ways, puppy raisers can be considered apprentices, receiving instructions provided by their organisations as they acquire puppy handling skills. Relevant to adult skills learning is the Cognitive Apprenticeship (CA) approach [54,55], which suggests developing apprentices’ ability to master tasks in a simulation setting before entering the real world, and that they may learn better with access to alternative methods when an initial approach fails to help. In the context of puppy raising, an opportunity for puppy raisers to be well-prepared by their organisation before obtaining the first puppy would be ideal but may be unrealistic to achieve, given the high demand for puppy raisers to start immediately, and the necessity of negotiating various developmental periods when raising a puppy. Short-term fostering opportunities may be of assistance. In addition, the reported benefits of having back-up sources of information deserve further research attention as this appears to accommodate preferences for different instructional methods.

The CA approach highlights a critical role for instructors in the learning process of apprentices, which often begins with a repeated demonstration of relevant tasks and then gradually fades to intensive supervision and active monitoring, and eventually less supervision and provision of support only when asked [55]. This process ends when the apprentice fully masters the required skills and can generalise to broader contexts and a range of situations. Regarding supervision in the context of assistance dog puppy raising, the current findings suggest that this activity generally involves routine visits from responsible personnel to meet directly with the puppy raisers regarding their puppy-raising practices. Frequent program supervision allowed staff to detect any undesirable behaviours appearing during the puppies’ psychological development. For instance, one puppy raiser (S14) maintained that “*proper follow up is super important. If a puppy gets sensitised (to a certain stimulus) and [staff] don’t notice it, it’s like a time that has been lost. So [it is helpful to have] proper follow up once a week.*”.

Intervals between check-up sessions varied among different organisations in the current study, from weekly to monthly or only a few times per year for those who lived far away from their organisation. During supervision, puppy raisers reported having the opportunity to practice relevant skills and receive feedback on how their puppy-raising practices adhere to the organisation’s guidelines. Considering the importance of adhering to strict rules as instructed by the organisation, supervision allows for staff to reward correct practices and advise when puppy raisers do otherwise. One program supervisor explained:

“*As far as following written instructions, I’m not impressed at all. We have them sign a checklist that says you will never allow this puppy to be off leash in an unenclosed area. And then people will post pictures on [online social media] of their puppy doing exactly that thing. So, if we put a rule into place but we don’t follow up or supervise them on that, over time they’re going to do whatever they want to do because they know it’s not going to be enforced.*”

(RS10)

While some raisers relied only on advice from their trainers, others preferred to seek help from those who had gone through the same situations. Overall, puppy raisers were positive about the opportunity to train and socialise their puppy with other puppy raisers. One puppy raiser stated:

“*You can read the puppy manual, you can read the timeline, you can get impressions on [social media] of what people are doing and what it should look like, but you don’t really know until you see other puppy raisers, and that those experienced puppy raisers might have a tip or two that really resonates with that first-time raiser and helps them out.*”

(RS10)

Program providers also acknowledged this source of informational support. One staff member explained why peer support might be helpful and what they have done to be resourceful:

“*I think the new raisers are very eager and they’re wanting to learn, and they’re very intrigued by the process, and you can’t cover everything. So, it’s just a matter of when you see something happening, explaining a different way of doing it. And then what we’re trying to do now, is when we have an experienced raiser, we try to buddy them up, so give them a mentor, that, if you have questions on how to do something, this is your mentor [when a] staff member isn’t available.*”

(S13)

To ensure appropriateness of advice offered by fellow puppy raisers, one puppy raiser (R03) said that “*there’s always a mediator in the group [who] would say, ‘okay this is not for fellow foster families to answer this, it is a matter of questions that must be directed to staff members.’*” While experienced puppy raisers would be able to support the more novice ones, for their own enquiries, they “*would then go more to the trainers, because they want to learn more and why this is happening*” (R03).

The informational supports that puppy raisers reported receiving from peers were in line with findings from research in educational settings [56,57,58]. Peer-teaching is one application of a scaffolded learning model [59], which highlights the importance of learning from those with higher ability but not exceeding the learner’s ‘zone of proximal development’ [60]. The zone of proximal development is when the demands of the tasks lie between the learner’s actual ability and a slightly higher but still achievable level of competency. As evident in the current study, experienced puppy raisers reported learning more from professional trainers while inexperienced ones would benefit greatly when learning from peers who were slightly more advanced than them. Organisations could also consider enhancing this peer learning process with support from suitably trained and accredited mediators to ensure their puppy raisers receive appropriate advice for their enquiries.

#### 3.2.2. Emotional Support

Besides formal and informal informational supports provided by program providers and other experienced puppy raisers, emotional support was necessary for puppy raisers, particularly novices. This type of support came from members of their organisation (e.g., staff and other puppy raisers) and those in existing networks (e.g., family and colleagues at their workplace).

One desirable attribute of staff was an ability to facilitate their helping relationships with puppy raisers. For instance, presenting themselves as being accessible, supportive and patient was reported as being what encouraged puppy raisers to elaborate further on difficult situations they were encountering. One puppy raiser said:

“*But more or less we’ve been having a regular weekly walk with [our dog trainer] with as many of us who can get there, with all these puppies, and that’s made a really big difference. Not only to the socialisation of the dogs, which has been a real bonus of that but as raisers being able to have these quiet conversations about you know, ‘Does your dog do this? Mine does this all the time. What do you do? This is terrible. Can’t bear it’ or positive stuff.*”

(R06)

Almost all puppy raisers and mentors reported that puppy raisers also benefited from emotional support provided by other puppy raisers in their organisation, both when dealing with puppies’ undesirable behaviours, and while grieving after returning their puppy to the organisation. One mentor/senior puppy raiser (RS12) attributed this to other puppy raisers’ experience and ability to relate to what they were going through. Puppy raisers reported that they tended to team up so that their socialising and training sessions would be more comforting as they could turn to someone else for help. Another mentor/senior puppy raiser explained:

“*[Other puppy raisers] who have done this before and can kind of show and answer some of the questions that are hard. Like how hard it is to return the dog and you know, what happens when that goes on. They can be very supportive of each other. That’s a good thing on [a social media platform] if they’re having problems or if their puppy does not make the program or that sort of thing. There’s a lot of good comforting comments that can be shared from older or from repeat raisers as well.*”

(RS10)

A few puppy raisers also received emotional support from people in the puppy raisers’ networks, such as friends or family members. One puppy raiser who lived alone shared their experiences of receiving support from neighbours for training their puppy. Others reported that family members were able to share the responsibility or help in some capacity, such as taking care of a puppy temporarily. One puppy raiser/mentor highlighted the importance of household members’ involvement in the puppy raising:

“*[Y]ou don’t want to be placing a puppy in a family where somebody [does not] want anything to do with this, you know, that always makes [it] harder on the raiser. So that the whole family kind of need to buy into the fact that you’re going to be raising the special puppy to be [an assistance dog] puppy.*”

(RS12)

Puppy raisers who were able to take their puppy to work reported positive experiences when receiving support from their workplace. Flexibility in workplace arrangements, such as working from home or allowing puppies at work, allowed puppy raisers to fulfil their responsibilities as a puppy raiser.

It appears that, during puppy raising, and especially during difficult times, puppy raisers benefit from different types of support (i.e., informational and emotional) and different sources of support (i.e., staff, fellow puppy raisers, family members, and colleagues). To highlight the importance of social support, one puppy raiser (RS12) repeatedly asserted that “it will take [a] village to raise the [assistance dog] puppies.” This finding is in line with the social support model proposed by Cohen and Wills [61], which holds that social support has two dimensions, functional and structural. The functional dimension includes instrumental support, such as financial aid and essential materials, and more intangible support, such as emotional support and information. Structural support, on the other hand, concerns the social connections of the individuals that provide different types of support. On this dimension, support can come from family, friends, organisations or social groups.

Regarding the reported positive effects of social support, the findings of the present study are in line with past research in human child-rearing [62,63], a practice which shares several similar characteristics with raising and educating a puppy. In the context of human child-rearing, a wide range of supports can be beneficial, and it is not easy to ascertain which support structures help with what issues specifically [62]. Social support, in general, is consistently found to act as a protective agent against parental burnout, depression, and stress [63,64,65]. The results presented in this paper suggest that this is also relevant to puppy raisers, with various types of social support, being provided by multiple sources, being a helping factor in enhancing the experiences of puppy raisers.

### 3.3. Puppy Characteristics

A third component that directly influences puppy-raising practices is the characteristics of the puppy itself. Puppies have been discussed in the assistance dog literature as the inputs (puppy breeding and selection) [66,67] and the products (puppies’ graduation and adult behaviours) [1,9,10] of the puppy-raising process, though little has examined how their characteristics influence this process. In the current study, puppy temperament was a factor affecting puppy raisers’ experience. A few puppy raisers reported that it would have been easier for their first-time puppy raising if the puppy had been less challenging. Other puppy raisers attributed having an easy-going puppy to their positive first-time puppy-raising experiences. One puppy raiser recalled:

“*I think the [puppy] had a really good personality for being [an assistance] dog because he was super calm everywhere, so I never had a problem when I took him to the subway or bus, there was no issue with him, he was just, ‘Okay, sit here’, and he was sitting and doing nothing, so in this way it was easy for me. And at home, I learnt [that] it’s a puppy, he was very playful.*”

(R04)

In addition to considering puppies’ different levels of difficulty, one participating program coordinator elucidated how they also consider physical factors of the puppy raisers when placing their puppies:

“*I’ve had a gentleman: a really tall, really deep voice, and older gentleman, and I wouldn’t give a really soft natured puppy to him because the deepness of his voice could accidentally frighten a softer-natured pup. So, the more outgoing ones would go there. My little old lady in a retirement village has got a small female puppy that’s just very laid-back and is absolutely thriving with her. Yeah, we do look at that when we’re placing pups.*”

(S15)

Puppy raisers appeared to prefer puppies whose behavioural demands were at levels they perceived they could manage. In the companion dog literature, undesirable characteristics, such as aggression, chasing, high energy, unfriendliness, distractibility, fearfulness, destructiveness, vocalisation, disobedience, soiling inside, jumping on people, and escaping [68] are the main reasons owners relinquish dogs to shelters. The former six have been explored in assistance dog research looking at breeding and selecting for suitable puppies [69,70,71]. However, to date, the latter six undesirable behaviours have not been studied in the assistance dog literature. In other words, puppy selection may filter out traits deemed unsuitable for public access; i.e., aggression, chasing and fearfulness, but may not reduce other traits associated with household-management issues. While puppies vary in their temperament and personality [72,73], the current findings suggest potential benefits to puppy raisers when matching puppies’ demands with puppy raisers’ level of experience, competency and their lifestyle.

## 4. Summary and Conclusions

**Figure 2:**
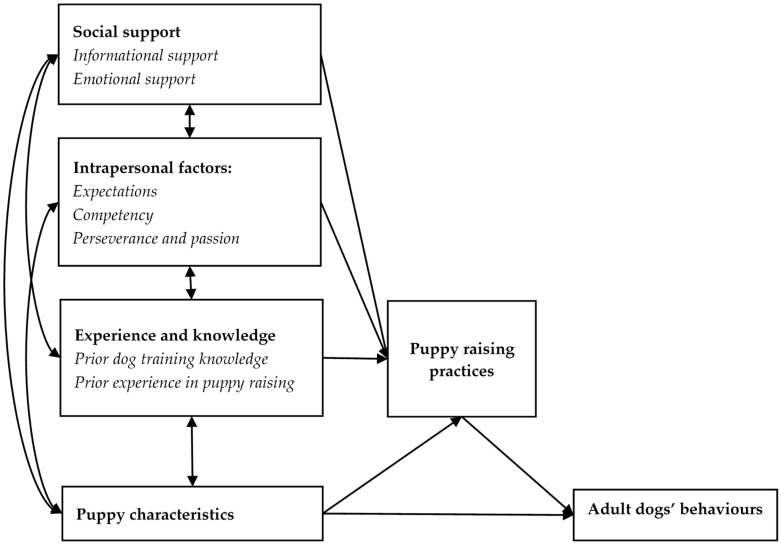
Proposed interrelations between various factors affecting puppy-raising practices.

Assistance dog puppy raising is generally a demanding job, especially for inexperienced puppy raisers. Previous research has suggested that experienced puppy raisers are more successful in puppy raising [6], but has yet to explain why. This study aimed to explore what helps and what hinders quality of assistance dog puppy raising. The results reveal three categories of factors that experienced puppy raisers and staff reported as affecting puppy-raising practices, namely *intrapersonal factors*, *social support* and *puppy characteristics*. Figure 2 illustrates proposed interrelations and effects of these factors on *puppy-raising practices*, which then directly influence puppies’ future behavioural outcomes. The intrapersonal factors include expectations, competency, passion and perseverance. These factors influenced puppy raisers’ perceived ability to raise puppies effectively, and whether they went on to raise more puppies and, hence, gained further experience and competency. This model extends upon the mediation model proposed in Figure 1 to suggest that, although experience contributes to raisers’ puppy-raising knowledge and competency, it is only one of several factors contributing to their overall puppy-raising practices. Social supports refer to different support types (informational and emotional), and sources of support (staff/organisation, other puppy raisers, family and friends) available to assist with puppy raising. While competency takes time and experience to develop (through perseverance), puppy raisers with low competency may benefit from receiving informational and emotional support from various sources when challenges arise. In addition, inexperienced raisers may find it more manageable to handle less-demanding puppies, or to practice puppy-raising skills with ‘easy’ puppies before engaging in their own puppy-raising practice.

Following on from the current findings, future research in assistance dog puppy raising should quantitatively measure the factors we identified at an individual level, such as puppy raisers’ competency and program engagement, and at a contextual level, such as overall supports received by puppy raisers. The contextual variables could then be correlated with puppy raisers’ practices and used to inform predictive models to establish which factors are most influential in determining puppies’ behavioural outcomes. While selecting competent and committed puppy raisers and less demanding puppies may be an ideal option, it is not always practical. Organisations may, therefore, need to take advantage of different sources and types of supports to ensure their puppy raisers have (1) realistic expectations of their puppy raising, (2) efficient learning and skills acquisition, and (3) sustained motivation to provide high-quality puppy-raising practices (see Table 2 for a summary of influencing factors and practical recommendations). If puppy-raising practices can be improved even marginally, such that fewer puppies fail to succeed, the potential benefits for assistance dog organisations and the clients and communities they service could be profound.

## Figures and Tables

**Table 1:** Participants’ roles and their experiences.

Code	Role	Country	No. Puppies Raised
R01	Puppy raiser	Australia	1
R02	Puppy raiser	Australia	2
R03	Puppy raiser	Canada	10
R04	Puppy raiser	Czech Republic	1
R05	Puppy raiser	Denmark	7
R06	Puppy raiser	New Zealand	1 *
R07	Puppy raiser	New Zealand	1
R08	Puppy Raiser	United States	20
SR09	Volunteer staff/puppy raiser	United States	0
RS11	Puppy raiser and staff counsellor/mentor	United States	18
RS10	Puppy raiser and staff-development supervisor	United States	18
RS12	Puppy raiser and staff counsellor/mentor	United States	23
S13	Staff—foster home coordinator	United States	–
S14	Staff—general manager	Mexico	–
S15	Staff—program coordinator	Australia	–
S16	Staff—program assistant	New Zealand	–
SRF17	Staff—raiser; founder, head trainer, and raiser of all puppies	United States	- **

* This participant had raised one puppy from the beginning until it was rehomed, followed by short-term raising of six other puppies. ** This person had bred and raised many puppies but could not specify the exact number.

**Table 2:** Summary of factors affecting puppy raising and recommendations for organisations.

Factors	Recommendations for Organisations
Intrapersonal factors	
*Expectations*. Having unrealistically high expectations of puppies’ training and behavioural development compromised positive experiences of puppy raisers.	Encourage realistic expectations through careful instruction and short-term experiential puppy raising.
*Competency*. Puppy-raising competency requires time and learning from prior experience to develop, and is critical in promoting favourable behavioural outcomes.	Provide prospective puppy raisers with information on canine behavioural development and puppy handling, as well as opportunities to practice training and socialisation skills under supervision.
*Barriers to help-seeking behaviour*. Puppy raising is challenging. Not knowing when to ask for help or perceiving judgements from others as negative hinders help-seeking behaviour.	Create a safe and non-judgemental environment to encourage help-seeking behaviours in puppy raisers, particularly the novice ones.
*Perseverance and passion*. Developing puppy-raising competency requires puppy raisers to persevere during times of difficulty, and positively appraise such perseverance.	Promote perseverance and passion by celebrating small achievements and ongoing contributions of puppy raisers.
Social support	
*Informational support*. Puppy raisers need to receive answers to inquiries promptly and in a form they prefer (e.g., in-person, written, or via telephone)	Provide accessible information and guidance from different sources and in different modalities. A qualified moderator may help redirect puppy raisers’ inquiries to those with relevant expertise.
*Emotional support*. Puppy raising is emotionally challenging at times, especially for inexperienced puppy raisers.	Establish puppy raisers’ support groups and involve family members and supportive others in the training and handling of puppies.
Puppy characteristics	
Puppies vary in their temperaments and behavioural characteristics. Some are more challenging than others. Puppy raisers with less experience may deal better with less challenging puppies.	Prioritise the placement of less-challenging puppies with novice puppy raisers. Puppies with behavioural issues (fearful avoidance, or high levels of energy) should be placed with puppy raisers with relevant competencies.

## Supplementary Materials

The following are available online at https://www.mdpi.com/2076-2615/10/1/128/s1: Table S1: Interview schedules for puppy raisers and staff.
