# Supplementary material for: Raising an Assistance Dog Puppy—Stakeholder Perspectives on What Helps and What Hinders
Source: Animals (Basel). 2020 Jan 13;10(1):128. doi: 10.3390/ani10010128 (PMC7023113; doi:10.3390/ani10010128)
Supplement: Supplementary file 1 [file animals-10-00128-s001.pdf]

Supplementary Material

Table S1. Interview schedules for puppy raisers and staff.

Puppy raiser participants	Staff participants
General questions	
- How many puppies have you raised so far?<br>- Tell me what it's like to be a puppy raiser.<br>- How would you compare your everyday life when raising your first puppy with your life before that?<br>- How would you compare the practice of raising an assistance dog puppy with raising a pet puppy?	- N/A
Motivations and expectations	
- What motivated you to become a puppy raiser in the first place?<br>- What were your expectations when you commenced the program?<br>- (If participants have raised more than one puppy), what motivated you to continue the practice of puppy raising?<br>- (If participants have raised more than one puppy) How would you compare your experience raising your first puppy with your experiences with subsequent puppies?	- What motivates people to become puppy raisers?<br>- What is required of a raiser to raise a successful puppy?<br>- Why do some raisers decide to raise another puppy, and others don't?
Insights	
- What do you think contributes to raising a successful puppy?<br>- What is required of you, and what are the ideal conditions for you to successfully raise an assistance dog puppy?	- What contributes to raising a successful assistance dog puppy?<br>- What are the ideal conditions to successfully raise an assistance dog puppy?
Challenges	
- Tell me about any challenges that you encountered.<br>- How did those difficulties affect your practice or the outcomes of your raising practice?<br>- What could've been done differently to eliminate those impacts?	- What kind of challenges do raisers commonly encounter?<br>- What do they do in those situations?<br>- What do you or [your organisation] do in those situations?
Puppy issues: health, behavioural issues	
- Did you experience any health or behavioural issues when raising your first puppy?<br>- Tell me what you felt when you encountered those issues<br>- What was your approach when the issue first happened?<br>- What did you do to search for a solution?<br>- Would you handle the situation differently now that you have had more experience?	- What are the issues that first-time raisers commonly have with their puppies?<br>- What do they usually do to respond to those situations?<br>- How about experienced raisers?

Table S1. Interview schedules for puppy raisers and staff (Cont.)

Puppy raiser participants	Staff participants
Training and engagement	
- Were you provided with clear instructions regarding your practice?<br>- Were these instructions realistic and how effective were you able to carry them out?<br>- How did your ability or inability to carry out the instructions affect your puppy outcomes?<br>- Could the organisation have provided any resources to help you better follow instructions?	- In terms of following instructions, how would you compare raisers' practice with what you expect them to do?<br>- To what extent does this affect the puppy outcomes?<br>- How different is it between first-time raisers and experienced raisers?<br>- What would make it more (or less) feasible for raisers to better follow instructions?
Knowledge	
- What do you know now that you wish you had known before you commenced your practice as a puppy raiser?<br>- What advice would you give to someone who was considering raising an assistance dog puppy for the first time?	- How often do raisers reach out to you to seek information about raising their puppy?<br>- How different is it between first-time raisers and experienced raisers?<br>- What could be done differently to better equip first-time raisers?
Organisational supports	
- What supports did your organisation offer to assist you as a puppy raiser?<br>- Could they have done anything more or different that would have improved your experience?	- What kind of supports do puppy raisers commonly seek from you [or your organisation]?<br>- What other types of supports would you like your organisation to offer, to better support the raisers?
Responsibility/workload	
- How do you feel about the workload?<br>How did the workload affect your practice, and how did you manage it?	- How do raisers feel about the workload of raising a puppy?<br>- What do you think about those perceptions?
